# The impact of challenge and hindrance demands on work-related burnout and job performance among employees in technology enterprises in China: the moderating effect of AI usage

**DOI:** 10.3389/fpsyg.2026.1811755

**Published:** 2026-08-18

**Authors:** Jing Zuo, Xuemei Sun, Zibai Wei

**Affiliations:** 1 Office of Academic Affairs, Nanning University, Nanning, China; 2 International College, National Institute of Development Administration, Bangkok, Thailand; 3 School of Accounting and Auditing, Guangxi Vocational Normal University, Nanning, China

**Keywords:** AI usage, challenge demands, hindrance demands, job performance, work-related burnout

## Abstract

**Background:**

Employees in technology enterprises face escalating job demands that profoundly shape their performance and wellbeing. While the Job Demands-Resources model and the Challenge-Hindrance Stressor Framework are well-established, the role of AI usage in moderating the stressor–performance pathway remains largely unknown, particularly in the high-pressure Chinese technology sector.

**Methods:**

Drawing on a sample of 442 employees from technology enterprises in Beijing, this study employed partial least squares structural equation modeling (PLS-SEM) to test a moderated mediation model examining the relationships among challenge demands, hindrance demands, work-related burnout, job performance, and AI usage.

**Results:**

Challenge demands positively predicted job performance (*β* = 0.297, *p* < 0.001), whereas hindrance demands negatively predicted it (*β* = −0.376, *p* < 0.001). Both types of demands increased work-related burnout (*β* = 0.410, *p* < 0.001; *β* = 0.514, *p* < 0.001), which in turn impaired performance (*β* = −0.407, *p* < 0.001). AI usage strengthened the positive effect of challenge demands on performance (*β* = 0.183, *p* < 0.001) and buffered the negative effect of burnout on performance (*β* = 0.252, *p* < 0.001), but did not mitigate the negative effect of hindrance demands on performance (*β* = 0.015, *p* = 0.729).

**Conclusion:**

This study extends the JD-R and CHSF frameworks by identifying AI as a dual-role job resource—enhancing the benefits of challenge demands while compensating for burnout-related resource depletion. However, AI cannot substitute for organizational interventions needed to address hindrance demands. These findings offer actionable guidance for strategically deploying AI alongside targeted organizational reforms to optimize employee performance.

## 1 Introduction

Employees in science and technology enterprises face intense job demands driven by rapid innovation and global competition (Deloitte, 2023b; OECD, 2021). Over 60% of technology workers report that their job demands exceed their coping capacity (Pandey, 2019)—a challenge particularly acute in China’s technology sector, where extended working hours and high-pressure conditions are prevalent (Wang et al., 2019). Given that job performance is critical to organizational success (Chen et al., 2019; Roczniewska et al., 2022), understanding how different types of job demands shape performance is both a scholarly priority and a managerial concern.

Two theoretical frameworks inform this inquiry. The Job Demands-Resources (JD-R) model (Demerouti et al., 2001; Bakker et al., 2023) proposes that job characteristics comprise demands and resources. Demands initiate a health-impairment process leading to burnout, while resources activate a motivational process enhancing engagement and performance (Bakker and Demerouti, 2007). The Challenge-Hindrance Stressor Framework (CHSF) (Cavanaugh et al., 2000; Podsakoff et al., 2023) extends this view by distinguishing two types of job demands. Challenge demands, such as high workload, time pressure, and responsibility, are appraised as opportunities for mastery and growth. Hindrance demands, such as role ambiguity, organizational politics, and administrative hassles, are appraised as obstacles that impede personal development and goal attainment (LePine et al., 2005; Podsakoff et al., 2023). These two types are theorized to have contrasting effects: challenge demands can motivate employees and improve performance, whereas hindrance demands tend to deplete resources and impair performance (Azeem et al., 2023; Van den Broeck et al., 2019). Integrating these frameworks allows us to examine whether challenge and hindrance demands exert differential effects on performance, directly and indirectly through burnout—a joint examination that neither framework alone fully captures.

Despite extensive research, several gaps remain. First, although the CHSF has received extensive scholarly attention, Podsakoff et al. (2023) called for more cross-cultural research on the framework. This call is especially pertinent to China’s technology sector, where employees face high workload and career pressures (Wang et al., 2019), yet the differential effects of challenge and hindrance demands on performance remain underexamined. Second, although burnout is theorized as a key mediator linking demands to performance (Crawford et al., 2010; Taris, 2006), prior research has not systematically compared whether challenge and hindrance demands impair performance through similar or distinct burnout-mediated pathways (cf. Baka et al., 2023). This leaves the differential mediating mechanisms largely unspecified. Third, the role of AI usage in moderating these relationships is largely unexplored. AI tools automate routine tasks, provide decision support, and facilitate information processing (Chen et al., 2021; Malik et al., 2022), suggesting that AI usage may function as a job resource within the JD-R framework (Liu and Li, 2025). Industry data show that 33% of employees in technology, media, and telecommunications regularly use AI, the highest adoption rate across all sectors (QuantumBlack, 2023). However, prior research has not systematically examined whether AI usage differentially moderates the effects of challenge and hindrance demands on burnout and performance. Specifically, it remains unclear whether AI usage can strengthen the positive effects of challenge demands, mitigate the negative effects of hindrance demands, or buffer the performance impact of burnout.

To address these gaps, this study integrates the JD-R model and CHSF to examine the relationships among challenge demands, hindrance demands, work-related burnout, job performance, and AI usage in Chinese technology enterprises. We propose and test a moderated mediation model in which work-related burnout mediates the effects of both types of demands on job performance, and AI usage moderates key paths in this process. We position AI usage as a novel job resource within the JD-R framework, one that may differentially alter the dynamics of challenge and hindrance stressors.

This study makes three contributions. First, it tests the integrated JD-R and CHSF frameworks in the context of China’s technology enterprises, extending their cross-cultural validity. Second, by modeling burnout as a mediator, it clarifies the psychological mechanisms linking both challenge and hindrance demands to performance. Third, it examines AI usage as a moderator within the JD-R/CHSF framework, revealing both its resource-enhancing and compensatory functions, as well as its boundary conditions in addressing hindrance demands. These findings offer practical insights for managers seeking to leverage AI for job design and employee performance.

## 2 Literature review

### 2.1 Theoretical foundations: integrating JD-R and CHSF

This study draws upon two complementary frameworks: the Job Demands-Resources (JD-R) model and the Challenge-Hindrance Stressor Framework (CHSF). The JD-R model (Demerouti et al., 2001; Bakker et al., 2023) categorizes job characteristics into demands and resources. Job demands require sustained effort and are associated with physiological and psychological costs; job resources help achieve work goals, reduce demands, or stimulate personal growth (Bakker and Demerouti, 2007). The model posits two parallel processes: a health-impairment process in which excessive demands deplete energy and lead to burnout, and a motivational process in which resources foster engagement and enhance performance (Bakker et al., 2023).

The CHSF (Cavanaugh et al., 2000; Podsakoff et al., 2023) refines this view by distinguishing two qualitatively different types of job demands. Challenge demands—such as high workload, time urgency, and responsibility—are appraised as opportunities for mastery and growth, triggering positive coping responses (LePine et al., 2005; Pindek et al., 2024). Hindrance demands—such as role ambiguity, organizational politics, and administrative hassles—are appraised as obstacles that consume energy without offering rewards, triggering negative emotional responses and disengagement (Podsakoff et al., 2023; Bao et al., 2024). Integrating these frameworks allows us to examine whether challenge and hindrance demands exert differential effects on job performance, directly and indirectly through burnout—a joint examination that neither framework alone fully captures.

### 2.2 Direct effects of challenge and hindrance demands on job performance

According to the CHSF, the two types of demands are theorized to have contrasting direct effects on performance. Challenge demands are appraised as opportunities for mastery and achievement (Cavanaugh et al., 2000). This positive appraisal activates the motivational process described in the JD-R model: employees perceive challenging demands as goals worth pursuing and mobilize cognitive, emotional, and behavioral resources to meet them (Bakker et al., 2023). Through this process, employees develop new skills, enhance self-efficacy, and improve task proficiency over time (LePine et al., 2005). Empirical evidence supports this reasoning. Azeem et al. (2023) found that challenge demands significantly enhanced job performance among Pakistani employees. A three-wave longitudinal study by Van Laethem et al. (2019) demonstrated a sustained positive impact on job performance in Finnish workplaces. In the Chinese context, Wang et al. (2021) found that challenge demands positively predicted job performance through enhanced positive emotions. Therefore, we propose:

*H1:* Challenge demands have a positive relationship with job performance.

In contrast, hindrance demands do not offer opportunities for growth or reward; instead, they thwart goal attainment (Podsakoff et al., 2023). According to the JD-R health-impairment process, such demands consume psychological resources without replenishing them, leading to frustration, disengagement, and reduced effort (Bakker and Demerouti, 2007). Resource depletion reduces cognitive capacity and motivation, directly impairing task performance (Crawford et al., 2010). Firdaus et al. (2023) found that high hindrance demands significantly reduced job performance among Indonesian bank employees. Mauno et al. (2020) reported that specific hindrance demands were associated with lower task performance. In the Chinese technology sector, hindrance demands—such as organizational politics and ambiguous role expectations—may be particularly detrimental to performance. Therefore, we propose:

*H2:* Hindrance demands have a negative relationship with job performance.

### 2.3 The mediating role of work-related burnout

While the direct effects above establish baseline relationships, both the JD-R model and CHSF suggest that job demands influence performance partly through employees’ psychological states—particularly burnout (Crawford et al., 2010). We therefore examine burnout as a mediator.

Challenge demands, despite being appraised as potentially rewarding, require sustained effort and energy expenditure (Podsakoff et al., 2023). Over time, cumulative strain from high workload and time pressure can deplete energy reserves, leading to emotional exhaustion—a core dimension of burnout (Crawford et al., 2010). Nair et al. (2020) found that challenge demands increased both work engagement and psychological burden, exacerbating burnout. Wu et al. (2020) demonstrated that excessive challenge demands could overwhelm Chinese technology employees’ coping resources, resulting in emotional exhaustion. Thus, even positive challenges, when sufficiently intense, can contribute to burnout. Therefore, we propose:

*H3:* Challenge demands have a positive relationship with work-related burnout.

Hindrance demands are more directly and consistently associated with burnout (Podsakoff et al., 2023). Perceived as unnecessary obstacles, they trigger negative emotions such as frustration and anxiety, consuming psychological resources without compensating gains (LePine et al., 2005). The JD-R health-impairment process predicts that such demands lead to resource depletion and eventually burnout (Bakker et al., 2023). Crawford et al. (2010) meta-analytically confirmed that hindrance demands are strongly and positively associated with burnout. In the Chinese context, Meng et al. (2023) found that hindrance demands—specifically interpersonal conflicts and organizational politics—significantly increased burnout. Therefore, we propose:

*H4:* Hindrance demands have a positive relationship with work-related burnout.

Burnout is characterized by emotional exhaustion, depersonalization, and reduced personal accomplishment (Halbesleben and Buckley, 2004). From the JD-R perspective, burnout represents the culmination of the health-impairment process: when energy resources are depleted, the capacity to focus, solve problems, and sustain effort is compromised (Bakker et al., 2023). This provides a clear theoretical link between burnout and performance decrements. Empirical research consistently supports this negative relationship. Abdul Aziz and Ong (2024) reported that burnout was associated with significantly lower performance in Southeast Asian workplaces. Lim et al. (2022) demonstrated that burnout negatively impacted project managers’ job performance in the Chinese construction sector. Moreover, burned-out employees tend to withdraw cognitively and emotionally, reducing innovation and collaboration—behaviors essential in technology enterprises (Yang and Hayes, 2020). Therefore, we propose:

*H5:* Work-related burnout has a negative relationship with job performance.

Taken together, H3–H5 imply that burnout mediates the effects of both types of demands on job performance. Challenge demands may have offsetting effects—enhancing performance directly but impairing it indirectly through burnout. Hindrance demands impair performance through both direct and burnout-mediated pathways. Therefore, we propose:

*H5a:* Work-related burnout mediates the relationship between challenge demands and job performance.

*H5b:* Work-related burnout mediates the relationship between hindrance demands and job performance.

### 2.4 The moderating role of AI usage

Within the JD-R framework, job resources help achieve work goals, reduce job demands, or stimulate personal growth (Bakker et al., 2023). Emerging evidence suggests that AI functions as such a resource: AI tools automate routine tasks, reduce cognitive load, and provide decision support, enabling more efficient and informed responses to job demands (Chen et al., 2021; Malik et al., 2022; Liu and Li, 2025). We therefore propose that AI usage moderates key pathways linking job demands and burnout to performance.

First, AI usage may strengthen the positive relationship between challenge demands and job performance. When employees face high challenge demands—such as heavy workloads and tight deadlines—AI tools help them manage these demands more efficiently. By automating low-value tasks, AI frees cognitive resources for high-value, creative work, allowing employees to maximize the motivational benefits of challenge demands while minimizing associated strain (Chen et al., 2021). Additionally, AI’s decision-support capabilities can enhance problem-solving and innovation, further amplifying the positive effects of challenge demands on performance. Therefore, we propose:

*H6:* AI usage moderates the relationship between challenge demands and job performance, such that the positive relationship is strengthened for individuals with higher AI usage.

Second, AI usage may weaken the negative relationship between hindrance demands and job performance. Hindrance demands derive much of their negative impact from uncertainty and lack of control—employees may not know what is expected or how to navigate organizational obstacles (Podsakoff et al., 2023). By providing transparent information and reducing ambiguity, AI may help employees plan and prioritize their work more effectively. Liu et al. (2026) demonstrated that when employees perceive AI as a challenge rather than a threat, it triggers positive motivational processes that help mitigate the negative effects of hindrance demands. However, because hindrance demands are inherently rooted in organizational and social dynamics—such as role ambiguity, interpersonal conflict, and opaque decision-making—rather than task-level inefficiencies, the buffering effect of AI may be limited if these obstacles stem from factors that technology alone cannot resolve. Nonetheless, we propose:

*H7:* AI usage moderates the relationship between hindrance demands and job performance, such that the negative relationship is weakened for individuals with higher AI usage.

Third, AI usage may buffer the negative relationship between burnout and job performance. The negative effect of burnout on performance is fundamentally driven by resource depletion: exhausted employees lack the energy to sustain high performance (Bakker et al., 2023). AI may act as a compensatory resource in this context. When burned-out employees use AI tools to automate routine tasks or receive decision support, they can maintain acceptable performance levels despite depleted energy. Additionally, AI’s ability to streamline workflows and reduce errors helps conserve remaining cognitive resources, mitigating the performance decline typically associated with burnout. Therefore, we propose:

*H8:* AI usage moderates the relationship between work-related burnout and job performance, such that the negative relationship is weakened for individuals with higher AI usage.

**Figure 1:**
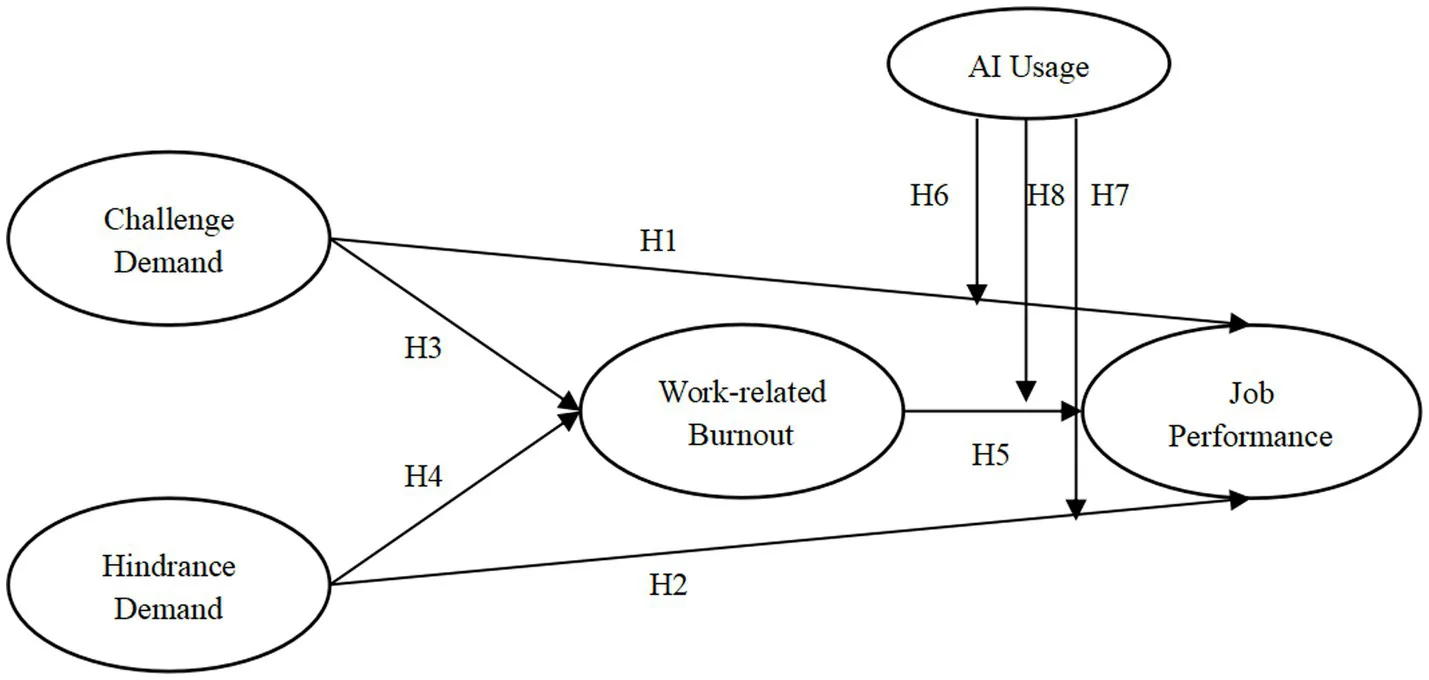
Proposed conceptual framework.

A conceptual model was built based on the hypotheses (see Figure 1).

## 3 Methodology

### 3.1 Population and samples

The target population comprises employees of science and technology enterprises in Beijing, specifically those in “Internet and Related Services” and “Software and Information Technology Services” (National Bureau of Statistics of China, 2023). Beijing was selected as the research site for its status as a leading technology hub with a diverse range of enterprises (Beijing Daily, 2023; Deloitte, 2023a) and a substantial workforce of 411,925 employees (EPS China Data, 2023), providing a robust sampling frame. The IT sector also exhibits one of the highest AI adoption rates (Capital Economics DCMS, 2022; QuantumBlack, 2023). We acknowledge that focusing on Beijing may limit generalizability to regions with different organizational cultures and AI adoption rates. The final sample (described below) comprised 442 valid responses, with a mean age of 29.28 years (SD = 7.60), 58.8% female, 66.97% ordinary employees, and 65.61% from medium/large firms (≥100 employees).

### 3.2 Data collection

After receiving institutional review board approval (ECNIDA 2023/0174), data were collected via a structured questionnaire distributed through the Credamo online survey platform from December 17, 2023, to January 28, 2024. Inclusion criteria were full-time employment in a Beijing-based technology enterprise with at least 6 months of tenure. Due to the unavailability of detailed employee lists, quota sampling was adopted, with quotas proportionally allocated based on industry subcategory and firm size. To mitigate common method bias, we assured anonymity, used different scale anchors for different constructs, and included attention-check items (Podsakoff et al., 2003).

Of 506 returned questionnaires, 442 were deemed valid after screening for attention checks, an AI screening question, and implausible completion time (less than 3 min). This exceeded the required sample size of 400, calculated using Taro Yamane’s formula (Yamane, 1973) with a 5% margin of error.

### 3.3 Measures

All constructs were measured using established scales. Except for challenge and hindrance demands, which used a 7-point scale (1 = not at all stressful to 7 = extremely stressful), all other measures employed a 7-point Likert scale (1 = strongly disagree to 7 = strongly agree). Following Brislin’s (1980) back-translation procedure, the questionnaire was translated into Chinese by a bilingual professor and back-translated by another professor proficient in English.

*Challenge and Hindrance Demands*. We used the 11-item scale by Cavanaugh et al. (2000): six items for challenge demands (e.g., “The number of projects and/or assignments I have”) and five for hindrance demands (e.g., “The degree to which my career seems ‘stalled’“). Cronbach’s alpha was 0.896 for challenge demands and 0.907 for hindrance demands.

*Work-Related Burnout*. We used the 6-item Copenhagen Burnout Inventory (Kristensen et al., 2005). A sample item is “How often do you feel tired?” Cronbach’s alpha was 0.920.

*Job Performance*. We used the Individual Work Performance Questionnaire (Koopmans et al., 2014), capturing task performance (five items) and contextual performance (five items). Sample items: “I was able to plan my work so that it was done on time” (task) and “I took on extra responsibilities” (contextual). Cronbach’s alpha was 0.879 for task performance and 0.886 for contextual performance.

*AI Usage*. We used three items adapted from Wang et al. (2023)’s AI Literacy Scale. Sample items include “I frequently use AI tools (e.g., ChatGPT, Copilot) in my daily work” and “I rely on AI to assist with complex tasks.” Cronbach’s alpha was 0.811.

*Control Variables*. We controlled for age, gender, marital status, job position, and firm size. Age was calculated from self-reported birthdate. Gender was coded 0 = male, 1 = female. Marital status 0 = unmarried, 1 = married. Job position 0 = ordinary employee, 1 = manager. Firm size 0 = fewer than 100 employees, 1 = 100 or more.

### 3.4 Data analysis

We used Partial Least Squares Structural Equation Modeling (PLS-SEM) via SmartPLS 4 (Ringle et al., 2024) to handle the complex model with multiple latent variables, mediation, and moderation (Zuo and Sun, 2024). The analysis followed the embedded two-stage approach for higher-order constructs (Sarstedt et al., 2019): Stage 1 assessed the lower-order measurement model for reliability (Cronbach’s *α*, composite reliability), convergent validity (average variance extracted, indicator loadings), and discriminant validity (Heterotrait-Monotrait ratio); Stage 2 assessed the higher-order construct (job performance) using lower-order component scores as indicators. The structural model was evaluated by examining path coefficients, bootstrapped significance levels (5,000 resamples), effect sizes (*f*^2^), and predictive relevance (*R*^2^ and *Q*^2^).

## 4 Results

### 4.1 The measurement model assessment of lower-order construct (LOC)

Following the embedded two-stage approach (Sarstedt et al., 2019), the first stage evaluates the lower-order measurement model for reliability and validity (Hair et al., 2023). As shown in Table 1, all indicator loadings exceeded 0.708 (Hair et al., 2021). Cronbach’s *α* ranged from 0.811 to 0.920, composite reliability from 0.886 to 0.938, and AVE from 0.657 to 0.728, all exceeding recommended thresholds and confirming satisfactory reliability and convergent validity. Table 2 presents the HTMT values, all below 0.90 for conceptually similar constructs and below 0.85 for conceptually different constructs, confirming discriminant validity (Hair et al., 2021).

**Table 1:** Reliability, validity, and factor loadings of lower-order constructs (LOC).

Constructs	*α*	CR	AVE	Items	Loadings
Task performance	0.879	0.912	0.674	Item1	0.804
				Item2	0.819
				Item3	0.822
				Item4	0.825
				Item5	0.834
Contextual performance	0.886	0.917	0.688	Item6	0.823
				Item7	0.802
				Item8	0.844
				Item9	0.830
				Item10	0.847
Challenge demand	0.896	0.920	0.657	Item1	0.841
				Item2	0.806
				Item3	0.790
				Item4	0.823
				Item5	0.815
				Item6	0.789
Hindrance demand	0.907	0.931	0.728	Item1	0.890
				Item2	0.849
				Item3	0.837
				Item4	0.860
				Item5	0.831
Work-related burnout	0.920	0.938	0.715	Item1	0.892
				Item2	0.850
				Item3	0.826
				Item4	0.832
				Item5	0.839
				Item6	0.832
AI usage	0.811	0.886	0.723	Item1	0.829
				Item2	0.883
				Item3	0.837

*α*, Cronbach’s alpha; CR, composite reliability and AVE, average variance extracted.

**Table 2:** Correlations, √AVE, and HTMT among constructs of the lower-order model (LOC).

Constructs	AI	WB	CD	CP	HD	TP
AI	0.850					
WB	0.112 (0.135)	0.845				
CD	0.125 (0.150)	0.491 (0.537)	0.811			
CP	0.110 (0.128)	−0.416 (0.460)	0.056 (0.072)	0.829		
HD	0.151 (0.181)	0.572 (0.626)	0.143 (0.158)	−0.478 (0.531)	0.854	
TP	0.092 (0.108)	−0.433 (0.482)	−0.014 (0.038)	0.600 (0.679)	−0.471 (0.527)	

AI, AI usage; TP, task performance; CP, contextual performance; CD, challenge demands; HD, hindrance demands; WB, work-related burnout; the square root of AVE is displayed diagonally; the value in brackets represents the HTMT ratio.

### 4.2 The measurement model assessment of higher-order construct (HOC)

For the higher-order construct (HOC), Job Performance, LOC scores were used as indicators in the second stage (Sarstedt et al., 2019). Table 3 shows that Cronbach’s *α*, CR, and AVE all exceeded recommended thresholds. Both task and contextual performance loadings were above 0.708 (Hair et al., 2021). Table 4 presents the intercorrelations and HTMT values, all below the recommended thresholds, confirming discriminant validity.

**Table 3:** Reliability, validity, and indicator loadings of the higher-order construct (HOC).

Constructs	*α*	CR	AVE	Loading
Job performance	0.750	0.889	0.800	
Task performance				0.893
Contextual performance				0.896

*α*, Cronbach’s alpha; CR, composite reliability and AVE, average variance extracted.

**Table 4:** Intercorrelations, √AVE, and HTMT among constructs of the higher-order model (HOC).

Constructs	AI	WB	CD	HD	JP
AI	0.850				
WB	0.112 (0.135)	0.845			
CD	0.125 (0.150)	0.491 (0.537)	0.811		
HD	0.151 (0.181)	0.572 (0.626)	0.143 (0.158)	0.854	
JP	0.113 (0.141)	−0.475 (0.572)	0.024 (0.051)	−0.531 (0.643)	0.894

AI, AI usage; JP, job performance; CD, challenge demands; HD, hindrance demands; WB, work-related burnout; the square root of AVE is displayed diagonally; the value in brackets represents the HTMT ratio.

### 4.3 Common method bias (CMB) assessment and multicollinearity

The marker variable approach was used to assess common method bias (Lindell and Whitney, 2001). All path coefficients associated with the marker variable were nonsignificant and close to zero, and their inclusion did not substantially alter the original path coefficients or significance levels, indicating that CMB was not a serious concern.

### 4.4 Structural model analysis

**Figure 2:**
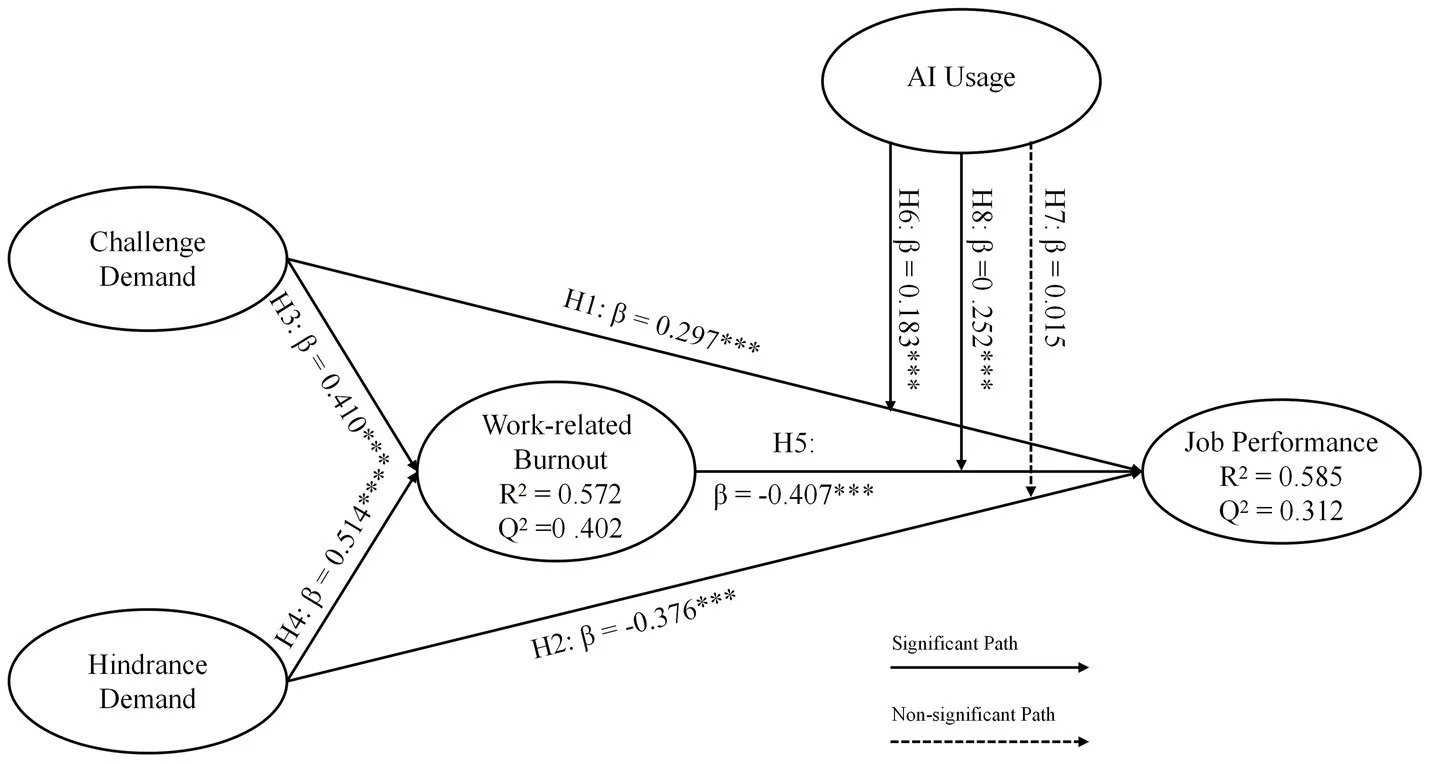
The results of the structural model. Notes: ^***^p < 0.001.

Table 5 and Figure 2 present the path coefficients and significance levels. Challenge demands positively predicted job performance (*β* = 0.297, *p* < 0.001, *f*^2^ = 0.145, medium effect), while hindrance demands negatively predicted it (*β* = −0.376, *p* < 0.001, *f*^2^ = 0.202, small-to-medium effect). Both challenge and hindrance demands positively predicted burnout (*β* = 0.409, *p* < 0.001, *f*^2^ = 0.381, large effect; *β* = 0.513, *p* < 0.001, *f*^2^ = 0.591, large effect), and burnout negatively predicted performance (*β* = −0.408, *p* < 0.001, *f*^2^ = 0.170, medium effect). *R*^2^ values for burnout (0.572) and performance (0.585) exceeded the 0.50 threshold for moderate explanatory power, and *Q*^2^ values (0.402 and 0.312) confirmed predictive relevance (Hair et al., 2019).

**Table 5:** Hypotheses testing.

Hypotheses	*β*	T Statistics	*p*	*f* ^2^
H1: Challenge demand → job performance	0.297	7.694	<0.001	0.145
H2: Hindrance demand → job performance	−0.376	8.792	<0.001	0.202
H3: Challenge demand → work-related burnout	0.410	12.620	<0.001	0.382
H4: Hindrance demand → work-related burnout	0.514	18.051	<0.001	0.591
H5: Work-related burnout → job performance	−0.407	8.052	<0.001	0.170

To test the mediating role of work-related burnout (H5a, H5b), we estimated indirect effects using bootstrapping with 5,000 resamples. As shown in Table 6, the indirect effect of challenge demands on job performance through burnout was significant (*β*_indirect = −0.174, SE = 0.025, 95% CI [−0.225, −0.128], *p* < 0.001), while the direct effect remained positive (*β*_direct = 0.297, *p* < 0.001), indicating competitive mediation (Zhao et al., 2010). The indirect effect of hindrance demands on job performance through burnout was also significant (*β*_indirect = −0.127, SE = 0.021, 95% CI [−0.171, −0.088], *p* < 0.001), with both direct and indirect effects negative, indicating complementary mediation. Thus, H5a and H5b are supported.

**Table 6:** Results of mediation effect analysis.

Path	Effect	*β*	SE	*p*	95% CI
CD → WB → JP	Direct	0.297	0.038	<0.001	[0.221, 0.372]
	Indirect	−0.174	0.025	<0.001	[−0.225, −0.128]
	Total	0.131	0.038	<0.001	[0.056, 0.201]
HD → WB → JP	Direct	−0.376	0.044	<0.001	[−0.460, −0.286]
	Indirect	−0.127	0.021	<0.001	[−0.171, −0.088]
	Total	−0.585	0.03	<0.001	[−0.643, −0.525]

CD, Challenge demands; HD, hindrance demands, WB, work-related burnout; JP, job performance.

### 4.5 Moderating effects

Table 7 presents the moderating effects. AI usage positively moderated the challenge demands–performance relationship (*β* = 0.183, *p* < 0.001), indicating that the positive effect of challenge demands on performance strengthens as AI usage increases. However, AI usage did not significantly moderate the hindrance demands–performance link (*β* = 0.015, *p* = 0.729). AI usage also positively moderated the burnout–performance relationship (*β* = 0.252, *p* < 0.001), indicating a buffering effect that weakens the negative impact of burnout on performance.

**Table 7:** Results of moderation effect analysis.

Path	*β*	Bootstrap 5,000 times			Percentile 95%	
		STDEV	*T*-stat	*p* values	Low	Upper
AI × CD → JP	0.183	0.039	4.624	<0.001	0.096	0.246
AI × HD → JP	0.015	0.043	0.347	0.729	−0.074	0.095
AI × WB → JP	0.252	0.050	5.025	<0.001	0.145	0.339

JP, job performance; CD, challenge demands, HD, hindrance demands, and WB, work-related burnout. AI, AI usage; JP, job performance; CD, challenge demands; HD, hindrance demands; WB, work-related burnout.

### 4.6 Robustness checks

Robustness checks were conducted for nonlinear effects, endogeneity, and unobserved heterogeneity (Sarstedt et al., 2020). Quadratic effect tests showed all path coefficients insignificant, indicating no nonlinearity. Gaussian copula tests showed no endogeneity concerns. For unobserved heterogeneity, FIMIX-PLS with segments from one to eight yielded varied metrics (AIC₃ indicated eight segments, CAIC two, BIC six). Following Sarstedt et al. (2020), such varied findings suggest that unobserved heterogeneity does not substantially influence the data.

## 5 Discussion

This study examined how challenge and hindrance demands affect job performance through burnout, and whether AI usage moderates these relationships. Most hypotheses were supported, except that AI usage did not significantly moderate the hindrance demands–performance link.

### 5.1 Direct and mediated effects of challenge and hindrance demands

Challenge demands positively predicted job performance, whereas hindrance demands negatively predicted it (H1, H2), consistent with CHSF’s opposing-effects proposition (Podsakoff et al., 2023; LePine et al., 2005). In the context of Chinese technology enterprises, employees appear to appraise high workload and time pressure as opportunities for growth and achievement, which stimulates effort and enhances performance (Azeem et al., 2023; Van Laethem et al., 2019). Conversely, hindrance demands such as role ambiguity and organizational politics deplete psychological resources without offering compensating gains, thereby impairing performance (Firdaus et al., 2023; Mauno et al., 2020).

Both types of demands increased burnout (H3, H4), which in turn reduced performance (H5), confirming burnout’s mediating role (H5a, H5b). This pattern aligns with the JD-R health-impairment process: excessive job demands—whether challenging or hindering—consume employees‘energy reserves, leading to exhaustion and reduced performance capacity (Bakker et al., 2023; Crawford et al., 2010). Notably, hindrance demands had a stronger effect on burnout (*β* = 0.514) than challenge demands (*β* = 0.410), suggesting that while challenge demands carry psychological costs, hindrance demands are more detrimental to wellbeing.

The mediation patterns differed between the two types of demands. Challenge demands exhibited competitive mediation: their direct positive effect on performance was partially offset by a negative indirect effect through burnout, reflecting the dual-edged nature of challenging stressors. Hindrance demands, in contrast, exhibited complementary mediation: both direct and indirect effects were negative, indicating a consistently detrimental influence. This divergence underscores the theoretical utility of distinguishing challenge from hindrance demands—their underlying mechanisms are qualitatively different, with challenge demands carrying both motivational benefits and psychological costs, whereas hindrance demands offer no compensatory advantages.

### 5.2 The moderating role of AI usage

AI usage significantly strengthened the positive challenge demands–performance relationship (H6: *β* = 0.183, *p* < 0.001). When employees use AI tools more frequently, they are better able to capitalize on challenging demands—such as high workload and tight deadlines—to enhance their performance. This finding supports the conceptualization of AI usage as a job resource within the JD-R framework (Liu and Li, 2025). By automating routine tasks and providing decision support, AI frees cognitive resources that employees can redirect toward strategic and creative problem-solving, thereby amplifying the motivational benefits of challenge demands (Chen et al., 2021).

AI usage also significantly buffered the negative burnout–performance relationship (H8: *β* = 0.252, *p* < 0.001), indicating that AI can serve as a compensatory resource for employees experiencing burnout. When burned-out employees use AI tools, they can maintain acceptable performance levels despite depleted energy—presumably because AI automates repetitive tasks and reduces the cognitive effort required to complete core work activities (Zhu et al., 2021). This is consistent with conservation of resources theory (Hobfoll, 1989): AI helps conserve what remains of employees’ psychological resources, mitigating the performance decline typically associated with burnout.

However, AI did not weaken the negative hindrance demands–performance relationship (H7: *β* = 0.015, *p* = 0.729). This null finding is theoretically informative. Hindrance demands—such as role ambiguity, organizational politics, and administrative hassles—are fundamentally rooted in organizational and social dynamics rather than task-level inefficiencies (Podsakoff et al., 2023). AI tools, however capable they are of automating tasks and providing data-driven insights, cannot resolve issues of unclear role expectations, interpersonal conflict, or opaque decision-making processes. This interpretation aligns with prior research suggesting that technology-based interventions are limited in their ability to address psychologically and socially complex workplace stressors (de Henestrosa et al., 2023; Schilbach et al., 2023). In essence, AI helps employees work around obstacles but cannot eliminate them—a critical boundary condition for AI’s workplace utility.

### 5.3 Theoretical contributions

This study makes three theoretical contributions. First, it extends the JD-R and CHSF frameworks to China’s technology sector, providing empirical evidence for their cross-cultural validity in a rapidly industrializing, high-pressure context. The findings confirm that the challenge–hindrance distinction and the health-impairment process operate similarly in this context as in Western settings, while also revealing the additional strain imposed by China’s intense technology-sector work culture. Second, it positions AI as a dual-role job resource—an enhancing resource that amplifies the positive effects of challenge demands, and a compensatory resource that mitigates the negative effects of burnout. This extends the JD-R model’s resource conceptualization, traditionally focused on interpersonal and organizational resources (e.g., social support, autonomy), to include technology-based resources operating at the task level. Third, the null moderation of AI on the hindrance demands–performance link refines the boundary conditions of AI’s utility: AI can help manage task-level demands but cannot resolve social, political, or structural stressors. Technology-based interventions complement, but do not substitute, organizational-level remedies such as role clarification and procedural fairness.

### 5.4 Practical implications

Our findings offer four practical implications. First, managers should use AI to automate routine tasks, freeing employees for complex work and amplifying the motivational benefits of challenge demands, with training and support essential for effective AI integration. Second, since AI cannot mitigate the negative effects of hindrance demands, managers must implement organizational measures—role clarification, procedural fairness, and transparent communication—to reduce these demands at their source; AI may assist in providing transparency, but it cannot resolve underlying interpersonal or structural issues. Third, AI can buffer burnout’s performance impact, but should not be viewed as a substitute for comprehensive burnout prevention strategies such as workload monitoring, flexible work arrangements, and access to psychological support. Fourth, managers should adopt a targeted AI strategy—deploying AI for task-related challenges and resource conservation, while complementing it with organizational development initiatives to address stressors rooted in organizational politics or role ambiguity.

### 5.5 Limitations and future directions

Several limitations should be acknowledged. First, the cross-sectional design precludes causal inferences; future research could use longitudinal designs. Second, the Beijing-based sample may limit generalizability; future studies could test the model in diverse cultural and sectoral contexts. Third, self-reported measures may be subject to social desirability and common method bias; although procedural remedies and the marker variable test indicated no substantial bias, future research could incorporate objective performance data or supervisor ratings. Fourth, we did not distinguish between different types of AI use (e.g., routine automation vs. creative ideation); future research could examine their differential moderating effects. Finally, the null finding for H7 raises important questions about AI’s boundary conditions; future qualitative research could investigate why AI appears unable to counteract hindrance demands.

## 6 Conclusion

This study examined how challenge and hindrance demands affect job performance through burnout, and whether AI usage moderates these relationships among employees in Chinese technology enterprises. Drawing on the JD-R model and CHSF, we tested a moderated mediation model with 442 employees.

Challenge demands positively predict job performance, while hindrance demands negatively predict it. Both types of demands increase burnout, which in turn impairs performance. AI usage strengthens the positive effect of challenge demands on performance and buffers the negative effect of burnout on performance, but does not mitigate the negative effect of hindrance demands. This suggests that AI functions as a job resource for task-related challenges and resource conservation, yet cannot resolve obstacles rooted in organizational or social dynamics.

These findings extend the JD-R and CHSF frameworks by incorporating AI as a novel job resource, and offer practical guidance for strategically deploying AI while addressing hindrance demands through organizational interventions.

## Data Availability

The raw data supporting the conclusions of this article will be made available by the authors, without undue reservation.

## References

[ref1] Abdul Aziz A. F. Ong T. (2024). Prevalence and associated factors of burnout among working adults in Southeast Asia: results from a public health assessment [original research]. Front. Public Health 12:1326227. doi: 10.3389/fpubh.2024.1326227 38550314 PMC10972957

[ref2] Azeem M. U. Haq I. U. Murtaza G. Jaffery H. (2023). Challenge–hindrance stressors, helping behavior and job performance: double-edged sword of religiousness. J. Bus. Ethics 184, 687–699. doi: 10.1007/s10551-022-05129-7

[ref3] Baka L. Prusik M. Jasielska D. (2023). Toward a better understanding of the health impairment process. Types of demand and burnout component matter. Front. Psych. 13:1037053. doi: 10.3389/fpsyt.2022.1037053, 36699490 PMC9868600

[ref4] Bakker A. B. Demerouti E. (2007). The job demands-resources model: state of the art. J. Manag. Psychol. 22, 309–328. doi: 10.1108/02683940710733115

[ref5] Bakker A. B. Demerouti E. Sanz-Vergel A. (2023). Job demands–resources theory: ten years later. Annu. Rev. Organ. Psych. Organ. Behav. 10, 25–53. doi: 10.1146/annurev-orgpsych-120920-053933

[ref6] Bao D. Mydin F. Surat S. Lyu Y. Pan D. Cheng Y. (2024). Challenge-hindrance stressors and academic engagement among medical postgraduates in China: a moderated mediation model. Psychol. Res. Behav. Manag. 17, 1115–1128. doi: 10.2147/PRBM.S448844, 38505350 PMC10949402

[ref7] Beijing Daily. (2023). Beijing reigns as top Chinese city in science and technology innovation development index. Available online at: http://bj.news.cn/2023-02/12/c_1129358903.htm (Accessed on November 12, 2023).

[ref8] Brislin R. W. (1980). “Translation and content analysis of oral and written material,” in Handbook of Cross-Cultural Psychology, eds. H. C. a. B. Triandis and J. W. (Boston: Allyn and Bacon), 389–444.

[ref9] Capital Economics DCMS. (2022). AI activity in UK businesses: executive summary. Available online at: https://www.gov.uk/government/publications/ai-activity-in-uk-businesses/ai-activity-in-uk-businesses-executive-summary (Accessed on October 03, 2023).

[ref10] Cavanaugh M. A. Boswell W. R. Roehling M. V. Boudreau J. W. (2000). An empirical examination of self-reported work stress among U.S. managers. J. Appl. Psychol. 85, 65–74. doi: 10.1037/0021-9010.85.1.65, 10740957

[ref11] Chen H. Li L. Chen Y. (2021). Explore success factors that impact artificial intelligence adoption on telecom industry in China. J. Manag. Anal. 8, 36–68. doi: 10.1080/23270012.2020.1852895

[ref12] Chen C.-X. Zhang J. Gilal F. G. (2019). Composition of motivation profiles at work using latent analysis: theory and evidence. Psychol. Res. Behav. Manag. 12, 811–824. doi: 10.2147/PRBM.S210830, 31565008 PMC6731976

[ref13] Crawford E. R. LePine J. A. Rich B. L. (2010). Linking job demands and resources to employee engagement and burnout: a theoretical extension and meta-analytic test. J. Appl. Psychol. 95, 834–848. doi: 10.1037/a0019364, 20836586

[ref14] de Henestrosa M. F. Sischka P. E. Steffgen G. (2023). Challenge, threat, coping potential: how primary and secondary appraisals of job demands predict nurses' affective states during the COVID-19 pandemic. Nurs. Open 10, 3840–3853. doi: 10.1002/nop2.1642, 36840623 PMC10170884

[ref15] Deloitte. (2023a). 2022 Deloitte China hi-tech high growth 50 and stars of tomorrow" list announced. Available online at: https://www2.deloitte.com/cn/zh/pages/technology-media-and-telecommunications/articles/2022-china-high-tech-star-list-announced.html (Accessed on January 18, 2024).

[ref16] Deloitte. (2023b). 2023 technology industry outlook. Available online at: https://www2.deloitte.com/content/dam/Deloitte/us/Documents/technology-media-telecommunications/2023-tmt-outlook-technology.pdf (Accessed on January 25, 2024).

[ref17] Demerouti E. Bakker A. B. Nachreiner F. Schaufeli W. B. (2001). The job demands-resources model of burnout. J. Appl. Psychol. 86, 499–512. doi: 10.1037/0021-9010.86.3.499, 11419809

[ref18] EPS China Data. (2023). China microeconomic data query system. Available online at: https://www.epsnet.com.cn/ (Accessed on December 06, 2023).

[ref19] Firdaus E. Z. Noermijati N. Ratnawati K. Zaroug Y. A. M. (2023). The role of job burnout and social support on the effect of job demand to employee performance. J. Appl. Manag. 21, 42–56. doi: 10.21776/ub.jam.2023.021.1.04

[ref20] Hair J. F. Hult G. T. M. Ringle C. M. Sarstedt M. Danks N. P. Ray S. (2021). Partial Least Squares Structural Equation Modeling (PLS-SEM) using R: A Workbook. Springer International Publishing.

[ref21] Hair J. F. Risher J. J. Sarstedt M. Ringle C. M. (2019). When to use and how to report the results of PLS-SEM. Eur. Bus. Rev. 31, 2–24. doi: 10.1108/EBR-11-2018-0203

[ref22] Hair J. F. Sarstedt M. Ringle C. M. Gudergan S. P. (2023). Advanced Issues in Partial Least Squares Structural Equation Modeling. Thousand Oaks, CA: Sage.

[ref23] Halbesleben J. R. B. Buckley M. R. (2004). Burnout in organizational life. J. Manag. 30, 859–879. doi: 10.1016/j.jm.2004.06.004

[ref24] Hobfoll S. E. (1989). Conservation of resources: a new attempt at conceptualizing stress. Am. Psychol. 44, 513–524. doi: 10.1037/0003-066X.44.3.513, 2648906

[ref25] Koopmans L. Bernaards C. M. Hildebrandt V. H. de Vet H. C. W. van der Beek A. J. (2014). Measuring individual work performance: identifying and selecting indicators. Work 48, 229–238. doi: 10.3233/WOR-131659, 23803443

[ref26] Kristensen T. S. Borritz M. Villadsen E. Christensen K. B. (2005). The Copenhagen burnout inventory: a new tool for the assessment of burnout. Work Stress 19, 192–207. doi: 10.1080/02678370500297720

[ref27] LePine J. A. Podsakoff N. P. LePine M. A. (2005). A meta-analytic test of the challenge stressor–hindrance stressor framework: an explanation for inconsistent relationships among stressors and performance. Acad. Manag. J. 48, 764–775. doi: 10.5465/amj.2005.18803921

[ref28] Lim S. Song Y. Nam Y. Lee Y. Kim D. (2022). Moderating effect of burnout on the relationship between self-efficacy and job performance among psychiatric nurses for COVID-19 in national hospitals. Medicina 58:171. doi: 10.3390/medicina58020171, 35208495 PMC8880477

[ref29] Lindell M. K. Whitney D. J. (2001). Accounting for common method variance in cross-sectional research designs. J. Appl. Psychol. 86, 114–121. doi: 10.1037/0021-9010.86.1.114, 11302223

[ref30] Liu Y. Li Y. (2025). Does human-AI collaboration promote or hinder employees’ safety performance? A job demands-resources perspective. Saf. Sci. 188:106872. doi: 10.1016/j.ssci.2025.106872

[ref31] Liu P. Yuan L. Tan Z. (2026). Proactive or defensive? How AI awareness influences employees' innovative behavior and knowledge hiding. Asia Pac. J. Hum. Resour. 64:e70072. doi: 10.1111/1744-7941.70072

[ref32] Malik A. Nguyen T. M. Budhwar P. (2022). Towards a conceptual model of AI-mediated knowledge sharing exchange of HRM practices: antecedents and consequences. IEEE Trans. Eng. Manag. 71, 13083–13095. doi: 10.1109/TEM.2022.3163117

[ref33] Mauno S. Kubicek B. Feldt T. Minkkinen J. (2020). Intensified job demands and job performance: does SOC strategy use make a difference? Ind. Health 58, 224–237. doi: 10.2486/indhealth.2019-0067, 31611468 PMC7286708

[ref34] Meng L. Du J. Lin X. (2023). Surviving bench stress: meaningful work as a personal resource in the expanded job demands-resources model. Curr. Psychol. 42, 17757–17768. doi: 10.1007/s12144-022-02956-9

[ref35] Nair A. V. McGregor A. Caputi P. (2020). The impact of challenge and hindrance demands on burnout, work engagement, and presenteeism. A cross-sectional study using the job demands-resources model. J. Occup. Environ. Med. 62, E392–E397. doi: 10.1097/jom.0000000000001908, 32404829

[ref36] National Bureau of Statistics of China. (2023). What categories are included in ISIC? Available online at: http://www.stats.gov.cn/zs/tjws/tjbz/202301/t20230101_1903769.html (Accessed on February 14, 2024).

[ref37] OECD. (2021). OECD science, technology and innovation outlook 2021. Paris: OECD Publishing. doi: 10.1787/75f79015-en

[ref38] Pandey J. (2019). Factors affecting job performance: an integrative review of literature. Manag. Res. Rev. 42, 263–289. doi: 10.1108/MRR-02-2018-0051

[ref39] Pindek S. Meyer K. Valvo A. Arvan M. (2024). A dynamic view of the challenge-hindrance stressor framework: a meta-analysis of daily diary studies. J. Bus. Psychol. 39, 1107–1125. doi: 10.1007/s10869-024-09933-y

[ref40] Podsakoff N. P. Freiburger K. J. Podsakoff P. M. Rosen C. C. (2023). Laying the foundation for the challenge–hindrance stressor framework 2.0. Annu. Rev. Organ. Psychol. Organ. Behav. 10, 165–199. doi: 10.1146/annurev-orgpsych-080422-052147

[ref41] Podsakoff P. M. MacKenzie S. B. Lee J.-Y. Podsakoff N. P. (2003). Common method biases in behavioral research: a critical review of the literature and recommended remedies. J. Appl. Psychol. 88, 879–903. doi: 10.1037/0021-9010.88.5.879, 14516251

[ref42] QuantumBlack. (2023). The state of AI in 2023: Generative AI’s breakout year. Available online at: https://www.mckinsey.com/capabilities/quantumblack/our-insights/the-state-of-ai-in-2023-generative-AIs-breakout-year (Accessed on February 05, 2024).

[ref43] Ringle C. M. Wende S. Becker J.-M. (2024). SmartPLS 4. Bönningstedt: SmartPLS.

[ref44] Roczniewska M. Smoktunowicz E. Calcagni C. C. von Thiele Schwarz U. Hasson H. Richter A. (2022). Beyond the individual: a systematic review of the effects of unit-level demands and resources on employee productivity, health, and well-being. J. Occup. Health Psychol. 27, 240–257. doi: 10.1037/ocp0000311, 34780212

[ref45] Sarstedt M. Hair J. F. Cheah J.-H. Becker J.-M. Ringle C. M. (2019). How to specify, estimate, and validate higher-order constructs in PLS-SEM. Australas. Mark. J. 27, 197–211. doi: 10.1016/j.ausmj.2019.05.003

[ref46] Sarstedt M. Ringle C. M. Cheah J.-H. Ting H. Moisescu O. I. Radomir L. (2020). Structural model robustness checks in PLS-SEM. Tour. Econ. 26, 531–554. doi: 10.1177/1354816618823921

[ref47] Schilbach M. Arnold M. Baethge A. Rigotti T. (2023). Hindrance demands as a boundary condition to the appraisal of challenge demands. Anxiety Stress Coping 36, 434–443. doi: 10.1080/10615806.2022.2108019, 35938551

[ref48] Taris T. W. (2006). Is there a relationship between burnout and objective performance? A critical review of 16 studies. Work Stress 20, 316–334. doi: 10.1080/02678370601065893

[ref49] Van den Broeck A. Van Hootegem A. Vander Elst T. De Witte H. (2019). Do self-enhancing and affiliative humor buffer for the negative associations of quantitative and qualitative job insecurity? Span. J. Psychol. 22:E8. doi: 10.1017/sjp.2019.7 30829182

[ref50] Van Laethem M. Beckers D. G. de Bloom J. Sianoja M. Kinnunen U. (2019). Challenge and hindrance demands in relation to self-reported job performance and the role of restoration, sleep quality, and affective rumination. J. Occup. Organ. Psychol. 92, 225–254. doi: 10.1111/joop.12239

[ref51] Wang Y. Li X. Xiao Z. (2019). Mental health of IT employees. Sci. Technol. Rev. 37, 45–52. doi: 10.3981/j.issn.1000-7857.2019.11.006 [in Chinese]

[ref52] Wang B. Rau P.-L. P. Yuan T. (2023). Measuring user competence in using artificial intelligence: validity and reliability of artificial intelligence literacy scale. Behav. Inform. Technol. 42, 1324–1337. doi: 10.1080/0144929X.2022.2072768

[ref53] Wang Q. Xia A. Zhang W. Cai Z. Zhang X. Teng X. . (2021). How challenge demands have offsetting effects on job performance: through the positive and negative emotions. Front. Psychol. 12:745413. doi: 10.3389/fpsyg.2021.745413 34970186 PMC8713591

[ref54] Wu H. Qiu S. Dooley L. M. Ma C. (2020). The relationship between challenge and hindrance stressors and emotional exhaustion: the moderating role of perceived servant leadership. Int. J. Environ. Res. Public Health 17:282. doi: 10.3390/ijerph17010282, 31906094 PMC6981731

[ref55] Yamane T. (1973). Statistics: An Introductory Analysis. 3rd Edn. New York: Harper & Row.

[ref56] Yang Y. Hayes J. A. (2020). Causes and consequences of burnout among mental health professionals: a practice-oriented review of recent empirical literature. Psychotherapy 57, 426–436. doi: 10.1037/pst0000317, 32463274

[ref57] Zhao X. Lynch J. G. Chen Q. (2010). Reconsidering baron and Kenny: myths and truths about mediation analysis. J. Consum. Res. 37, 197–206. doi: 10.1086/651257

[ref58] Zhu X. Wang S. He Q. (2021). Impact of skill requirements on employees’ thriving at work: from the perspective of artificial intelligence embedding. Foreign Econ. Manag. 43, 15–25. doi: 10.16538/j.cnki.fem.20210330.106

[ref59] Zuo J. Sun X. (2024). Impact of challenge and hindrance demands on work-related burnout: the mediating effect of psychological empowerment. J. Behav. Sci. 19, 16–29. Available at: https://so06.tci-thaijo.org/index.php/IJBS/article/view/27174

